# Prediction Signatures in the Brain: Semantic Pre-Activation during Language Comprehension

**DOI:** 10.3389/fnhum.2016.00591

**Published:** 2016-11-15

**Authors:** Burkhard Maess, Fahimeh Mamashli, Jonas Obleser, Liisa Helle, Angela D. Friederici

**Affiliations:** ^1^MEG and Cortical Networks Group, Max Planck Institute for Human Cognitive and Brain SciencesLeipzig, Germany; ^2^Department of Neuropsychology, Max Planck Institute for Human Cognitive and Brain SciencesLeipzig, Germany; ^3^Department of Neurology, Massachusetts General Hospital, Athinoula A. Martinos Center for Biomedical Imaging, Harvard Medical SchoolBoston, MA, USA; ^4^Max Planck Research Group “Auditory Cognition”, Max Planck Institute for Human Cognitive and Brain SciencesLeipzig, Germany; ^5^Department of Psychology, University of LübeckLübeck, Germany; ^6^Elekta OyHelsinki, Finland; ^7^Department of Neuroscience and Biomedical Engineering, School of Science, Aalto UniversityEspoo, Finland

**Keywords:** semantics, prediction, language, MEG, N400

## Abstract

There is broad agreement that context-based predictions facilitate lexical-semantic processing. A robust index of semantic prediction during language comprehension is an evoked response, known as the N400, whose amplitude is modulated as a function of semantic context. However, the underlying neural mechanisms that utilize relations of the prior context and the embedded word within it are largely unknown. We measured magnetoencephalography (MEG) data while participants were listening to simple German sentences in which the verbs were either highly predictive for the occurrence of a particular noun (i.e., provided context) or not. The identical set of nouns was presented in both conditions. Hence, differences for the evoked responses of the nouns can only be due to differences in the earlier context. We observed a reduction of the N400 response for highly predicted nouns. Interestingly, the opposite pattern was observed for the preceding verbs: highly predictive (that is more informative) verbs yielded stronger neural magnitude compared to less predictive verbs. A negative correlation between the N400 effect of the verb and that of the noun was found in a distributed brain network, indicating an integral relation between the predictive power of the verb and the processing of the subsequent noun. This network consisted of left hemispheric superior and middle temporal areas and a subcortical area; the parahippocampus. Enhanced activity for highly predictive relative to less predictive verbs, likely reflects establishing semantic features associated with the expected nouns, that is a pre-activation of the expected nouns.

## Introduction

Language comprehension is a demanding process, which requires decoding of highly structured speech signals within very short time (Friederici, 2002; Pylkkänen and Marantz, 2003; Poeppel et al., 2008). Success of speech perception is increased through the combination of input-driven and prediction-based analysis (Strauß et al., 2013). Predictions are derived from both prior knowledge and contextual information (Rao and Ballard, 1999; Bar, 2007; Bendixen et al., 2009; Griffiths and Tenenbaum, 2011). These predictions are needed to achieve an optimal performance, both in general low level sensory processing as well as in higher cognitive processing such as speech perception. They do not stretch into some far future, they rather concern the present. According to predictive coding theory, our brain continuously anticipates current sensory input by transferring information from hierarchically higher to lower areas via top-down processing (Engel et al., 2001; Friston, 2003; Bar, 2009; Kiebel et al., 2009; Huang and Rao, 2011; Rauss et al., 2011). This reduces the processing demands at lower levels of hierarchy if the input matches expectations. If the prediction was wrong, performance will be less than optimal, as appropriate actions for the predicted input might have started already. However, in the case of weak or no predictions, no negative influence on the performance is to be expected.

Recently, modeling language processing using predictive coding theory has gained attention (David et al., 2011; Hickok, 2012; Sohoglu et al., 2012; Jakuszeit et al., 2013; Park et al., 2015; Lewis et al., 2016). Gagnepain et al. (2012) propose a predictive coding model of single spoken word recognition and show that superior temporal gyrus (STG) neurons represent the difference between predicted and heard speech sounds. Interestingly, instead of a direct competition between co-activated words they claim that alternative word completions compete via eliciting different predictions. Furthermore, researchers now also focus on the neural mechanism related to “predictive” processing itself (i.e., the context that generates the prediction). In a visual paradigm using magnetoencephalography (MEG) measurements Dikker and Pylkkänen (2013) found increased activation in left middle temporal and left inferior frontal gyrus (IFG) to pictures of objects (e.g., a picture of an apple) that were predictive for a specific word (e.g., apple) compared to pictures that were not predictive for the same word (e.g., a picture of shopping bag full of groceries). Fruchter et al. (2015) investigated the serial processing of adjective-nouns phrases using MEG and observed higher activity in the left middle temporal gyrus (MTG) in response to highly predictive adjectives in adjective-noun phrases. Using combined eye-tracking and functional magnetic resonance imaging (fMRI), Bonhage et al. (2015) revealed the neural substrate for syntactic word category prediction in a distributed network including cortical areas relevant for language processing as well as subcortical areas.

So far, predictions in language processing have been reported for many linguistic levels such as lexical, syntactic and sentential semantics (Kutas and Federmeier, 2000; DeLong et al., 2005; Bonte et al., 2006; Dambacher et al., 2006; Federmeier, 2007; Dikker and Pylkkanen, 2011; Obleser and Kotz, 2011; Van Petten and Luka, 2012; Brusini et al., 2015; Lewis and Bastiaansen, 2015). Previous findings suggest that all contextual information have an immediate impact on linguistic predictions (Hale, 2001; Demberg and Keller, 2008; Levy, 2008; Smith and Levy, 2013). Particularly, N400 responses were robustly found as being sensitive to variations in lexical-semantic prediction (Kutas and Hillyard, 1980, 1989; Federmeier, 2007; Lau et al., 2009, 2013a,b). It has been reliably observed that N400 amplitude is reduced following a predictive or supportive context (Kutas and Hillyard, 1984; Federmeier et al., 2007; Kutas and Federmeier, 2011; Wlotko and Federmeier, 2012).

The goal of the current study was the investigation of semantic context building during sentence processing. The main idea is that highly predictive verbs contribute more than average information towards the overall sentence meaning while the less predictive verbs provide less than average and keep a lot of options open. Consequently, the processing of the predicted nouns mainly constitutes of a confirmation whereas the processing of the less predicted nouns still involves at least the retrieval of semantic information and integration into the preceding context. While prediction formation has been investigated in the context of reading, thus far evidence from speech is less well-established. For this aim, we designed an MEG study based on a sentential N400 paradigm. In simple, spoken subject-verb-object sentences of German, we varied the predictability relation between the context (verb) and a sentence’s final word (noun) as being high (e.g., *He drives the car*) or low (e.g., *He gets the car*). We used identical final determiner-noun phrases together in both contexts. Following highly predictive verbs, the presented noun is the one with highest cloze probability (Taylor, 1953). Following less predictive verbs, the (same) noun is one of many alternatives, however, all alternatives had equally low (or even lower) cloze probabilities. Consequently, none of our nouns is violating a prediction from the preceding context.

So far, using MEG, several approaches were taken in identifying the brain network underlying the N400m. In summary, the results suggest a left hemispheric dominance and the involvement of temporal and inferior frontal sources (Halgren et al., 2002; Marinkovic et al., 2003; Pulvermüller et al., 2005; Maess et al., 2006; Pylkkänen and McElree, 2007; Salmelin, 2007; Dikker and Pylkkänen, 2013). In addition, previous N400 studies also reported activity of deeper structures during the processing of semantics; these were perirhinal and inferior temporal structures active in semantic aspects of word recognition (Halgren et al., 2006) as well as thalamic nuclei responding specifically to semantic violations (Wahl et al., 2008).

We hypothesize that the previously shown reduction of the N400 for the highly expected words is mostly a consequence of the prior prediction formation during the processing of earlier presented semantic context. Less expected words need more processing effort and therefore produce a larger evoked response, because, more semantic information needs to be retrieved. Violations of the preceding context are not discussed here as such words may cause a more or less extensive error handling which are special cases in speech processing.

## Materials and Methods

### Stimuli

**Figure 1:**
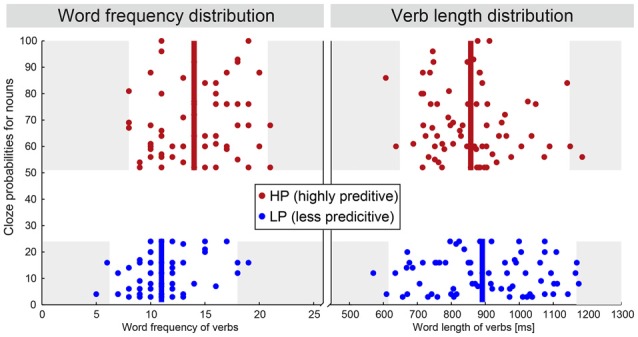
**Distribution of the word frequency and word length values for the verbs in both conditions.** Both differences are small and therefore considered irrelevant for the effects discussed here. For more details see “Materials and Methods” Section—Stimuli. The white area marks the centered 95% percentile.

We selected short German sentences in which the verb was either highly predictive (e.g., *he drives the car, German: Er fährt das Auto*) or less predictive (e.g., *he* *gets* the car, German: Er kriegt das Auto) of the following noun. Stimuli were taken partially from an earlier study (Gunter et al., 2000) and an additional behavioral pre-study. In both cases participants had to fill in a printed form which provided a personal pronoun (Er/Sie [he/she]) followed by a verb. Participants were asked to complete the sentence beginnings in a simple way by providing just a determiner and a noun. During the analysis, we identified pairs of verbs falling in either of the two classes—highly predictive (cloze probability >50%) or less predictive (cloze probability <25%) of the following noun as previously done by Gunter et al. (2000). For the purposes of the present study, all stimuli were additionally controlled for their word frequencies^1^ and word length (Figure 1). The complete set of stimuli consists of 69 pairs. Sentences could be interpreted literally—a figurative interpretation is unlikely. As we use the same noun phrase for each of the sentence pairs—there are no stimulus driven differences at the noun level between both conditions. The verbs, however and intentionally, consist of two different groups. The median word frequency between both verb groups is different by a value of 3 which corresponds to a ratio 1:8. Halgren et al. (2002) observed a minor influence of word frequency towards the N400 when comparing mean word frequencies of 15 with 336 per million, which corresponds to a ratio of about 1:23. We have analyzed a subset of 39 pairs of our stimuli, which had no difference in the median word frequency nor in word length. We did not observe a change in the evoked condition differences between both stimulus sets. The set with 69 pairs had a higher signal-to-noise ratio though; hence we based the following analysis on the complete set of stimuli. Sentences were spoken by a trained female speaker at natural speed. Loudness was adjusted so that all sentences were at the same perceived level.

### Participants

In total, 21 German native speakers (11 female) took part in an MEG experiment (age range: 20–32 years, median: 27). All were right-handed according to the Edinburgh Handedness Inventory (Oldfield, 1971). None of them had a hearing deficit or neurological diseases. Participants gave written informed consent prior to the experiment and were paid for their participation. The study follows the guidelines of the declaration of Helsinki and has an ethical approval of the ethics commission of the University of Leipzig.

### Design and Procedure

**Figure 2:**
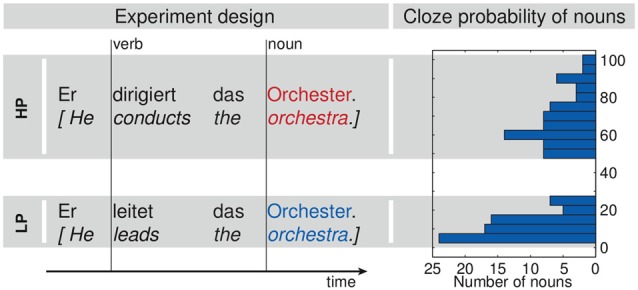
**Experimental design.** The left part shows two exemplary sentences out of the set of 69 pairs. Vertical lines mark the acoustic onsets of the verbs and nouns as used for the analysis. The histogram at the right shows the distribution of the cloze probability values for the nouns in both conditions.

Participants were seated in a dimly lit shielding room (Vacuumschmelze Hanau, Germany). MEG data was recorded with a 306 channel Vectorview device (Elekta Oy, Helsinki, Finland) at 500 Hz sampling rate utilizing a bandwidth of 160 Hz. Additionally, three bipolar channels were recorded to monitor eye and cardiac activity. Electrooculogram (EOG) electrodes were attached to the outer canthi and above and below the left eye. Electrocardiogram (ECG) electrodes were placed at the right clavicle and the left ribs. The experiment was conducted in one session with five recording blocks. First, participants’ individual hearing thresholds were determined for both ears separately. Stimuli were presented at 48 dB SL (sensation level, i.e., above the individually determined hearing threshold). Sensation levels were estimated using a subpart of one sentence. Stimuli were randomized and presented once over two blocks, thereafter presentation of all stimuli was repeated in two further blocks using a different randomization. The onsets of all sentences, as well as the onsets of the verbs and the nouns in each sentence were specifically marked (Figure 2). During the fifth block participants heard a sequence of sinusoidal tones. Data of this block are not presented here.

Participants were instructed to listen carefully to the presented sentences and not to move during a block. They were asked to fixate their view at a cross at the center of the screen while listening to the auditory stimulation. Fixation crosses were presented from 700 ms before onset until 700 ms after the offset of each sentence. Fifteen percent of the sentences were followed by either the same or an alternative sentence spoken by a different voice (male). Participants’ (incidental) task was to judge whether the two succeeding sentences (female and male voice) were the same. Participants responded by a button press following the cue. The cue also informed participants of the response-to-button-alignment: it comprised of one happy and one sad symbolic face (smiley), one presented on either side of the screen. Participants had to use their thumbs to press either the left or the right side button, i.e., giving the answer “yes” with the button at the side of the happy face and “no” with the other. The arrangement of the faces on the screen was randomized and counterbalanced over all stimuli in each block.

### Data Preprocessing

**Figure 3:**
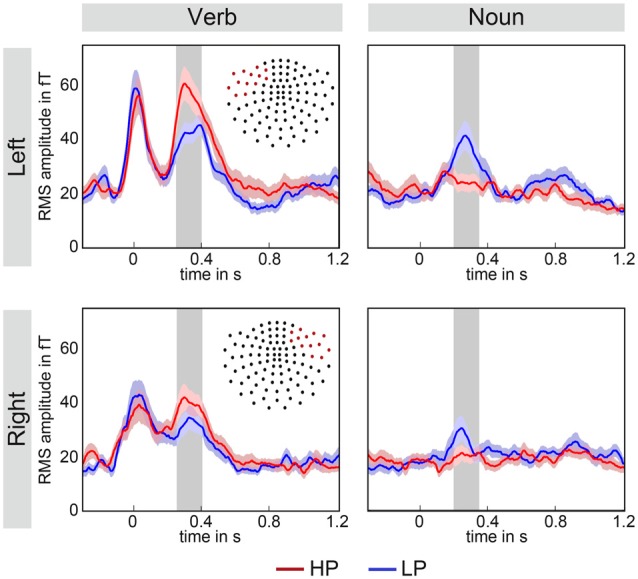
**Grand averaged root mean squared (RMS) signals of two fronto-temporal magnetometer sensor groups averaged over all participants.** The upper row shows left hemisphere data and the lower row right hemisphere data. Note, that each red dot in the channel layout represents the location of the selected magnetometers. The time interval of interest is marked by the gray area. Left and right channels show a similar pattern, namely that the HP (red) over LP (blue) effect for verbs is reversed for nouns LP (blue) over HP (red). This difference appears to be more expressed over the left hemisphere. The color-shaded areas around the time courses represent the standard-error-of-the-mean as determined by the across subjects averaging.

MEG data were corrected for head movements and external interferences by the Signal Space Separation (SSS) method (Taulu et al., 2005) implemented in MaxFilter^TM^ software (Elekta Oy). Additionally, a recently developed SSS-based method (Taulu et al., 2012) for suppression of the uncorrelated sensor noise and artifacts was applied prior to SSS-processing to further enhance the signal-to-noise ratio. The MEG signals were filtered (0.2–9 Hz) to focus on the evoked, broad N400 deflection, as similarly done in e.g., (Maess et al., 2006; David et al., 2011). The high-pass filter (4367 points FIR, hamming window) was specifically designed for a strong DC suppression (>125 dB at DC) to replace the baseline correction. High-pass filtering was used instead of the classical baseline correction, because there is no “clean” time interval in the middle of a sentence when using normal connected speech as stimulus. Data was subjected to a spatial independent component analysis (ICA) decomposition using MNE python (Gramfort et al., 2013). ICA components, which showed a high temporal correlation with EOG or ECG channels and with a typical topography, were manually identified and removed before reconstructing the MEG signals. Two to five ICA components were excluded with a median of 3 over all subjects and blocks. Subsequently, epochs were selected as 1500 ms intervals (from −300 ms to 1200 ms with respect to the onset of verbs or nouns, respectively). Epochs during which the standard deviation of signal channels within a sliding 200 ms time window exceeded either 200 pT/m, 5 pT or 150 μV (for gradiometers, magnetometers and EOG, respectively) were excluded from further analysis. Epochs were averaged into four categories: highly predictive verbs, less predictive verbs, highly predicted nouns and less predicted nouns. For simplicity, we use the same acronyms when labeling verb and noun conditions: HP means either highly predictive or highly predicted when used as either verb or noun condition label. LP (less predictive/predicted) is the counterpart. Preprocessing was performed using the MNE toolbox (Gramfort et al., 2014) and own Matlab^2^ routines. Evoked fields were subjected to source localization using the MNE toolbox (Gramfort et al., 2014) and finally mean sLORETA estimates of a total of nine selected cortical regions were estimated. Cluster analyses (Maris and Oostenveld, 2007) were conducted to justify the selections of the channels and the interval in time (see Figure 3). Evoked responses for the verbs and the nouns over all magnetometers and over the complete time interval from −300 ms to 1200 ms were subjected to two cluster analyses comparing HP vs. LP. Since condition (HP/LP) is a within-subjects-variable—a two-sided paired *t*-test (“depsamplesT”) was used for the generation of clusters (α-threshold = 5%). Clusters “clusterstatistic = maxsum; minnbchan = 2” were statistically tested using Monte-Carlo-randomizations (*N* = 5000, “correctm = cluster”) and a statistical threshold of 1%.

### Source Localization

Source localization of the evoked responses was performed using sLORETA minimum-norm estimation (Hämäläinen and Ilmoniemi, 1994; Pascual-Marqui, 2002), implemented in MNE Software^3^. sLORETA for cortically constrained surfaces results in noise-normalized activity values (*z*-values). The sign of this sLORETA solution equals the sign of the current density estimated as minimum norm estimate at the same vertex. sLORETA regularization was set to an SNR value of 3. Co-registration was based on the digitized head shape and the fiducial points. Individual anatomical T1 weighted MR images were segmented using Freesurfer 5.1.0^4^. Based on this segmentation, single compartment boundary element models (BEM) were constructed and source spaces were provided as a set of current dipoles located within and mostly perpendicular to the cortical surfaces (loose value = 0.2, which means that the source covariance matrix entries which correspond to the current surface normal are set equal to one and the two others are set equal to 0.2). Furthermore, individual cortical surfaces were morphed onto the cortical surface of one participant to allow group averaging of the sLORETA estimates (Dale et al., 1999). ROIs were selected as subparts of regions defined by a Freesurfer parcellation (Destrieux et al., 2010). We selected nine regions per hemisphere known as parts of the network for semantic language processing (Lau et al., 2008; Price, 2010; Friederici, 2011), namely the primary auditory cortex (PAC), the planum polare (PP), the planum temporale (PT), the anterior and posterior parts of the MTG (aMTG and pMTG), the temporal pole (TP), the IFG including Brodmann areas BA44 and BA45 and the parahippocampal gyrus aPH and pPH. ROI magnitudes were computed as the mean of the absolute sLORETA values within each of the pre-selected ROIs. Two-way ANOVAs were conducted for each of the nine ROIs with the factors HEM (hemisphere) and PRED (predictability).

## Results

### Sensor-Level Analysis of The N400 Effects

For sensor-level group analysis, the root mean squared (RMS) signal of a group of channels over left and right fronto-temporal regions was computed (see Figure 3). RMS signals were estimated for gradiometers and magnetometers and for the verbs and the nouns, separately. Time range and channel selection were made in accordance with current literature which identified early, fronto-temporal N400 components when using MEG, see for instance (Pylkkänen et al., 2002; Maess et al., 2006). Analysis of the responses to the nouns (see Figure 3, right column) showed, as expected, a significant N400m effect observed for both sensor types—we present the values for the magnetometers only (LP-HP Left: *p* < 0.001, *t*_(20)_ = −4.25; Right: *p* < 0.05, *t*_(20)_ = −2.07) in the time window 200–350 ms with a larger amplitude for less predicted nouns. Most interestingly, at the preceding verbs (see Figure 3, left column) a significantly larger amplitude was observed for highly predictive compared to less predictive verbs (Left: *p* < 0.001*, t*_(20)_ = 4.26; Right:* p* = 0.005*, t*_(20)_ = 3.15) in the time window 250–400 ms. Note that the time intervals, represented as gray areas in the figures, for the verb and noun tests were equal in length, but shifted by 50 ms towards earlier latencies for the nouns.

**Figure 4:**
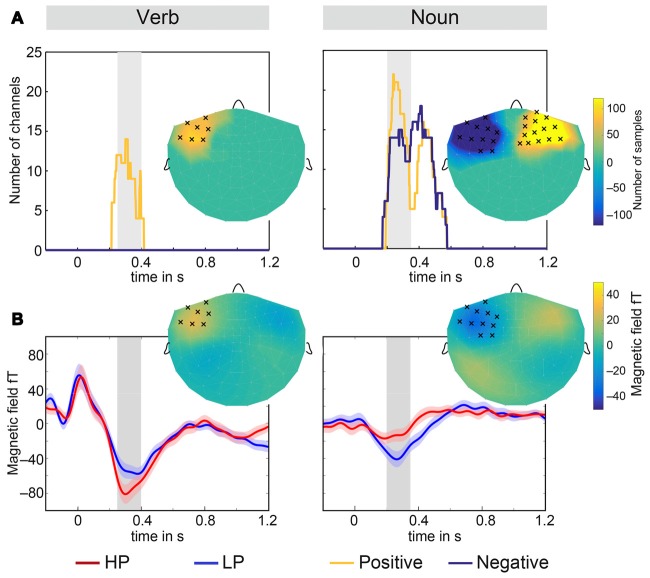
**Results of the cluster analysis. (A)** Spatio-temporal extent of the three clusters for verbs (left) and nouns (right). The gray bar in the background marks the temporal intervals which were used throughout this article. The inserts show topographically the full number of samples in a cluster. Channels which had 50 or more cluster samples within the gray intervals were marked by crosses. **(B)** Average time courses of left hemispheric magnetometers in clusters for verbs and nouns and both conditions LP and HP. The shaded area around the solid lines displays the standard error of the mean. The inserts show topographically the magnetic field difference LP-HP averaged over the time interval marked in gray. Marked channels have 50 or more cluster samples within the gray interval.

Cluster analyses were conducted for an independent justification of the selection of channels and time interval (Maris and Oostenveld, 2007). For the verbs, significant positive magnetic field differences were observed for two intervals in time—the first roughly located between 1000 ms and 1200 ms and the second between 250 ms and 400 ms. The average length of the verbs is about 900 ms (see Figure 1), therefore only the earlier interval is relevant for the processing of verbs. This cluster mainly included left fronto-temporal channels (see Figure 4A left). No significant effect for negative differences (right hemisphere) were observed. For the nouns, a positive and a negative cluster were observed both stretching in time from about 200 ms–500 ms (see Figure 4A right). Clusters included both left and right fronto-temporal channels. Therefore, the selection of channels and time interval in the RMS signal *t*-tests was highly overlapping with the cluster analyses results. Average time courses of significant channels within a cluster in the left hemisphere as well as the magnetic field difference (LP-HP) topography for verbs and nouns is shown in Figure 4B.

Sensor analysis in general is prone to a head-position bias: if heads were systematically closer to the left side of the dewar then sensors at this side also showed stronger responses. We therefore refrain from formulating arguments concerning the left-right differences of the above analysis and rather refer to source space analysis instead. Source analysis included the complete set of MEG sensors (gradiometers and magnetometers) and is therefore independent of the RMS channel selection or the channels in clusters, respectively.

### Source Localization of The N400 Effects

**Figure 5:**
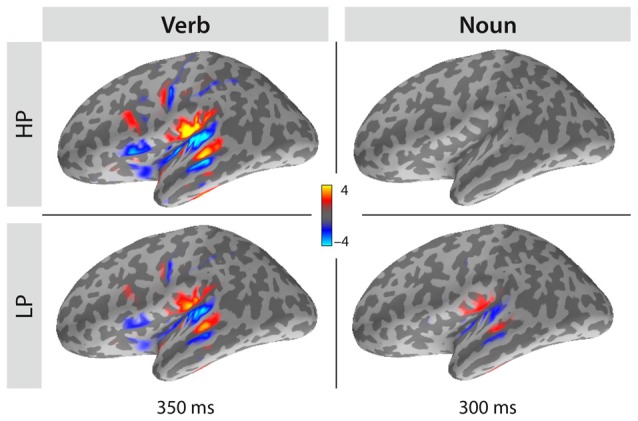
**Grand average sLORETA minimum norm source localization results projected to surface normals for both conditions (HP and LP) and both word classes (verb and noun) computed for latencies of 350 ms or 300 ms, respectively.** Note, that the distribution of estimates is spatially very equivalent although it differs in strength. Interestingly, there is a reversal between conditions: highly predictive verbs (HP) elicit stronger responses than less predictive verbs (LP), but less predicted nouns (LP) elicit a stronger response than highly predicted nouns (HP).

To compare the spatial distributions of the sLORETA estimates for both the verb and the noun time intervals, the signed sLORETA estimate was additionally computed: here the projection to the surface normals of the folded cortex replaces the magnitudes. Individual estimates were then morphed onto the cortex of one of our participants. Figure 5 displays the grand-average (mean across subjects) for the two conditions (HP and LP) at two latencies (350 ms for verbs, 300 ms for nouns). Three of the four panels show larger, left temporal estimates. Note, that the polarity and the location of the estimates are very comparable between all three panels. These single condition panels demonstrate that conditions mainly differ in strength, but not in spatial distribution. Computing the difference LP-HP (not displayed in the figure), which is the classical N400 in case of the nouns, leads to a similar picture as shown in the lower right panel. Computing LP-HP for the verbs, leads to a polarity reversal as HP has the stronger estimates compared to LP but otherwise very similar spatial distribution. We interpret this observation such that sentence processing asks for a certain amount of computational demands, which can be delivered at either the verb level, when the verb is highly predictive, or at the noun level, at the latest. Since the activity at the verb level is overall stronger than activity at the noun level, we would argue that recognizing a verb and setting up a meaningful context for it, needs some extra processing demands not needed at the following noun level. This is plausible as verbs may additionally generate specific syntactic restrictions, i.e., objects with special case markings. Less predictive verbs must provide less information than highly predictive verbs as the first do not allow specifying the next word. Lesser information, lesser processing demands, hence we expect a smaller evoked response for them. The same holds for highly predicted nouns which do not add much new information, because they are predicted—therefore their processing is finished early by about 250 ms.

**Figure 6:**
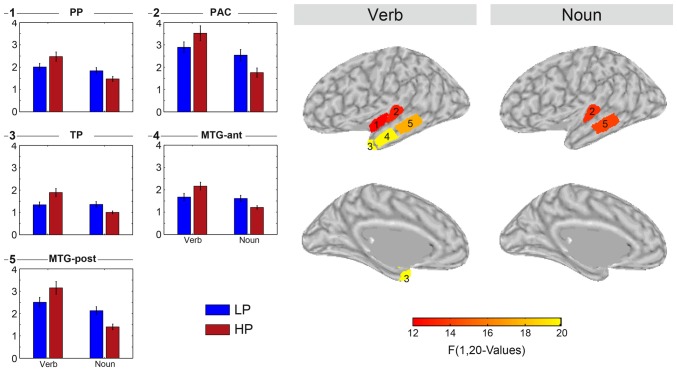
**Results of separate two-way ANOVAs testing the mean sLORETA estimate of each of the nine ROIs (HEM × PRED).** Displayed are the five ROIs which showed a significant main effect of PRED. The displayed *F*-values represent significant effects (*p* < 0.05) after Bonferroni correction.

Mean sLORETA estimates were computed for each of the nine regions in both hemispheres as the mean over all estimates within a region. Figure 6 left panels (bar graphs) display the sLORETA estimates for those regions which showed a significant main effect of the condition (HP vs. LP) for both word classes (verb, noun). The bar graphs also show the standard error of the mean as a short black line at the top of each bar. Note that the sign of the difference always reverses when comparing the verb and the noun condition difference. Figure 6 right panels visualize the location and extent of each region with a significant effect overlaid of lateral or medial views of the inflated left hemisphere.

**Figure 7:**
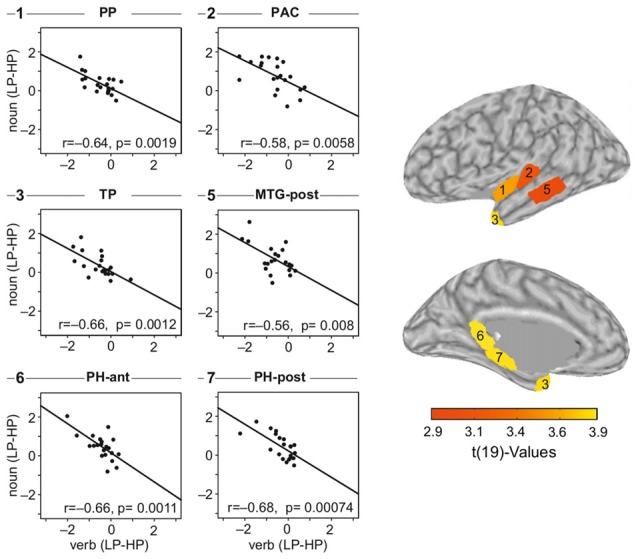
**Correlation plots for the six ROIs which showed a significant relation between the verb- and the noun-effect.** Effect sizes are estimated as the difference between the mean sLORETA estimates of the two conditions: LP-HP. The underlying data (before computing the difference) was just positive (magnitudes). The difference switches signs as HP elicited larger amplitudes during verb processing and LP during noun processing.

Figure 7 visualizes the across-participants correlation between the verb and the noun effect, that is difference LP-HP for both intervals. The dots in the scatter plots in the two left columns present single participant data. The line is estimated via linear regression of the verb and the noun effect. We have computed the regression for each of the nine cortical regions separately and in Figure 7, we show the data of those six regions in which a significant correlation was observable. Raw *p*-values are given within each of the panels. All regions, but pMTG and PAC are significant when correcting for multiple comparisons by factor of 18 (Bonferroni). pMTG and PAC have *p*-values of 0.14 and 0.11 after correction, respectively.

## Discussion

The neural signature of prediction during language comprehension was investigated using MEG sensor level analysis and sLORETA estimates. These revealed the expected large N400 amplitude for the less predicted (unexpected) nouns compared to predicted (expected) nouns; very consistent with previous studies using similar sentences and electroencephalography (EEG) or fMRI measurements (Gunter et al., 2000; Hald et al., 2007; Obleser and Kotz, 2010; Wang et al., 2012). Interestingly, when focusing on the prior verb we observed a reverse pattern: stronger N400 responses for the highly predictive (i.e., informative) vs. the less predictive verbs. Crucially, we found a negative correlation between the verb N400 effect and the noun N400 effect in left temporal cortex (PAC, PP, TP and pMTG) and also left parahippocampal area, suggesting an underlying functional relation.

The sLORETA estimates for the verbs and the nouns were very similar (Figure 5) pointing to a similarity in the functional domain. The classical N400 observed for unpredicted nouns and the N400 effect for the predictive verbs are both expressions of lexical-semantic processes indicated by a high correlation between the two. The observed N400 effect for the verbs did not depend on the integration of prior context, as we had provided just a minimal context of randomly using either of two personal pronouns (Sie vs. Er [She vs. He]). The verb N400 may thus represent differences in the semantic retrieval accounting for the differences in information for the two verb groups as a function of predictive power. Magnetic field and localized source topographies show that verbs and nouns activated a very similar network of semantic processes. So, one functional interpretation of the verb N400 may reflect a pre-activation of conceptual features due to semantic relatedness (Federmeier and Kutas, 1999; Van Petten et al., 1999; Kutas and Federmeier, 2000). In models of semantic memory, it is considered that there is a link between those items that share perceptual and functional features in common (Kutas and Federmeier, 2000; Patterson et al., 2007; Coutanche and Thompson-Schill, 2015). There is ample empirical evidence that mental representation of the verbs also include detailed grammatical and semantic information of the nominal components related to the verb (e.g., object; Shapiro et al., 1987, 1993; Friederici and Frisch, 2000; Li et al., 2006; Thompson et al., 2007). Therefore, semantic information of the verb impose selectional restrictions about the arguments, such as whether the arguments are animate or inanimate, human or nonhuman, instruments or non-instruments (Li et al., 2006). According to high-dimensional semantic space models derived from large-scale corpus analyses, such as Hyperspace Analogue to Language (HAL; (Burgess and Lund, 1997) and Latent Semantic Analysis (LSA; Landauer and Dumais, 1997), a word is defined by reference to the other words that co-occur with it. These co-occurrences can be encoded as a vector of weightings. Highly predictive verbs likely have strong weightings with a few specific co-occurrence items; consequently, less predictive verbs should have only weak co-occurrence weightings to other words. We assume that predictions are only made if evidence is sufficiently high. Therefore, processing of the predictive verbs likely lead to a co-activation of expected nouns, however, less predictive verbs do not cause multiple predictions for all possible continuations. Consequently, we hypothesize that during sentence processing the benefit of a confirmed prediction is paid earlier in time by stimulating the retrieval of semantic information. This is supported by the inverse correlation between the verb and the noun N400 effects. Forming predictions during sentence comprehension should be seen as a side effect of establishing semantic meaning while listening to the auditory input. If the recognized words allow a more rigid setup of the context, then this context is established immediately thereby creating a more restrictive prediction for the words to follow and thereby yielding an overall performance increase.

In terms of the source localization, the verb induced N400 effect demonstrated increased activation for the highly predictive vs. the less predictive verbs in mostly left hemispheric regions. Left hemispheric dominance was reported as well in another MEG study focusing on reading nouns in predictive contexts for latencies beyond 150 ms, especially for the non-matching nouns (Dikker and Pylkkänen, 2013). More specifically, our results demonstrated a network of temporal and parahippocampal regions to be involved in prediction related processing of the N400 effect. In contrast to the neural sources found for a classical N400 effect, we did not observe left IFG to be part of the predictive processing network. Left IFG has been found important for selection of lexical candidates (Thompson-Schill et al., 1997, 1999; Lau et al., 2008). Therefore, one could have expected to observe a negative correlation between verb and noun N400 effects in the left IFG, if the predictive process was considered to be a controlled selection process. However, our data did not show involvement of the left IFG for predictive processing suggesting that the observed process was rather based on automatic co-occurrences than on a controlled process. For the left anterior temporal cortex, our results are consistent with Lau et al. (2016) who found this area to be most relevant for top-down facilitation of lexical-semantic predictions. Our results are also in line with those of Bonhage et al. (2015) who observed distributed temporal sources and no IFG activity for word specific predictions using fMRI. Finally, a prominent predictive effect was found in parahippocampal regions, which has been reported for linguistic processing (Meyer et al., 2005; Tracy and Boswell, 2008; Duff and Brown-Schmidt, 2012) and for predictive processing in general (Schiffer et al., 2012). Taken together, we interpret the fact that areas known to be involved in the classical N400 generation were also involved in predicting upcoming nouns as indicative for predictive coding (Bonhage et al., 2015).

The present results may allow us to formulate a simplified version of the predictive coding model on the N400 generation: let’s presume that the processing of word B (e.g., *car*) depends on whether the preceding context word A is highly predictive (*A*_p_; e.g., *drive*) or not (*A*_n_; e.g., *get*). The predictive coding model assumes that *A*_p_ (e.g., *drive*) generates a prediction 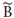 for the word B (e.g., *car*) with connections conveying 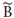 from higher to lower cortical hierarchies. Processing of word B (e.g., *car*) would then require only the forward processing of the difference B-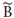 up the processing hierarchy. The smaller the difference between prediction 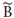 and actual input B (e.g., *car*), the lesser bottom-up information transfer and lesser adjustment at the higher level is required. In contrast, when word *A*_n_ (e.g., *get*) does not allow generating predictions 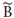, all information in word B (e.g., *car*) has to be conveyed from lower to higher levels. In summary, the processing of word B (e.g., *car*) and the internal information transfers much depend on the processing of the preceding context word A (e.g., *drive* or *get*), its specific information content and its relation to the target word B (e.g., *car*).

## Conclusion

In summary, in the present sentence comprehension experiment we demonstrate that processing of predictive verbs as compared to less predictive verbs, evokes a response which is very similar to the classical N400 response in its temporal and distributional parameters. The observed N400 was larger for the predictive (i.e., informative) verbs than for the less predictive verbs. The predictive-verb N400 and the less predicted-noun N400 was inversely correlated which demonstrates a direct trade-off in terms of neural expenditure between the predictive and the predicted stage in lexical-semantic domain during language comprehension. The finding that evoked responses by the verbs were overall stronger than those by the nouns appears to reflect that, in general, verbs carry more information than nouns, thereby supporting the view that the N400 amplitude matches the amount of processed information. Our suggestion for a generalized functional interpretation of the N400 effect is thus that it reflects the effort to establish semantic meaning from perceived information.

## Author Contributions

BM and FM: experiment design, data collection, data analysis, article preparation; JO and ADF: experiment design, data analysis, article preparation; LH: data analysis, article preparation.

## Conflict of Interest Statement

The authors declare that the research was conducted in the absence of any commercial or financial relationships that could be construed as a potential conflict of interest. The reviewer ES and handling Editor declared their shared affiliation, and the handling Editor states that the process nevertheless met the standards of a fair and objective review.

## Footnotes

^1^http://wortschatz.uni-leipzig.de/

^2^http://www.mathworks.com/

^3^www.nmr.mgh.harvard.edu/martinos/userInfo/data/MNE_register

^4^surfer.nmr.mgh.harvard.edu

## References

[B1] Bar M. (2007). The proactive brain: using analogies and associations to generate predictions. Trends Cogn. Sci. 11, 280–289. 10.1016/j.tics.2007.05.005 17548232

[B2] Bar M. (2009). Predictions: a universal principle in the operation of the human brain. Introduction. Philos. Trans. R. Soc. Lond. B Biol. Sci. 364, 1181–1182. 10.1098/rstb.2008.0321 19527998 PMC2666718

[B3] Bendixen A. Schröger E. Winkler I. (2009). I heard that coming: event-related potential evidence for stimulus-driven prediction in the auditory system. J. Neurosci. 29, 8447–8451. 10.1523/JNEUROSCI.1493-09.2009 19571135 PMC6665649

[B4] Bonhage C. E. Müller J. L. Friederici A. D. Fiebach C. J. (2015). Combined eye tracking and fMRI reveals neural basis of linguistic predictions during sentence comprehension. Cortex 68, 33–47. 10.1016/j.cortex.2015.04.011 26003489

[B5] Bonte M. Parviainen T. Hytönen K. Salmelin R. (2006). Time course of top-down and bottom-up influences on syllable processing in the auditory cortex. Cereb. Cortex 16, 115–123. 10.1093/cercor/bhi091 15829731

[B6] Brusini P. Brun M. Brunet L. Christophe A. (2015). Listeners exploit syntactic structure on-line to restrict their lexical search to a subclass of verbs. Front. Psychol. 6:1841. 10.3389/fpsyg.2015.01841 26696917 PMC4678230

[B7] Burgess C. Lund K. (1997). “Representing abstract words and emotional connotation in a high-dimensional memory space,” in Proceedings of the Cognitive Science Society, (Hillsdale, NJ: Erlbaum), 61–66.

[B8] Coutanche M. N. Thompson-Schill S. L. (2015). Creating concepts from converging features in human cortex. Cereb. Cortex 25, 2584–2593. 10.1093/cercor/bhu057 24692512 PMC4537422

[B9] Dale A. M. Fischl B. Sereno M. I. (1999). Cortical surface-based analysis. I. Segmentation and surface reconstruction. Neuroimage 9, 179–194. 10.1006/nimg.1998.0395 9931268

[B10] Dambacher M. Kliegl R. Hofmann M. Jacobs A. M. (2006). Frequency and predictability effects on event-related potentials during reading. Brain Res. 1084, 89–103. 10.1016/j.brainres.2006.02.010 16545344

[B11] David O. Maess B. Eckstein K. Friederici A. D. (2011). Dynamic causal modeling of subcortical connectivity of language. J. Neurosci. 31, 2712–2717. 10.1523/JNEUROSCI.3433-10.2011 21325540 PMC3384564

[B12] DeLong K. A. Urbach T. P. Kutas M. (2005). Probabilistic word pre-activation during language comprehension inferred from electrical brain activity. Nat. Neurosci. 8, 1117–1121. 10.1038/nn1504 16007080

[B13] Demberg V. Keller F. (2008). Data from eye-tracking corpora as evidence for theories of syntactic processing complexity. Cognition 109, 193–210. 10.1016/j.cognition.2008.07.008 18930455

[B14] Destrieux C. Fischl B. Dale A. Halgren E. (2010). Automatic parcellation of human cortical gyri and sulci using standard anatomical nomenclature. Neuroimage 53, 1–15. 10.1016/j.neuroimage.2010.06.010 20547229 PMC2937159

[B15] Dikker S. Pylkkanen L. (2011). Before the N400: effects of lexical-semantic violations in visual cortex. Brain Lang. 118, 23–28. 10.1016/j.bandl.2011.02.006 21458057

[B16] Dikker S. Pylkkänen L. (2013). Predicting language: MEG evidence for lexical preactivation. Brain Lang. 127, 55–64. 10.1016/j.bandl.2012.08.004 23040469

[B17] Duff M. C. Brown-Schmidt S. (2012). The hippocampus and the flexible use and processing of language. Front. Hum. Neurosci. 6:69. 10.3389/fnhum.2012.00069 22493573 PMC3319917

[B18] Engel A. K. Fries P. Singer W. (2001). Dynamic predictions: oscillations and synchrony in top-down processing. Nat. Rev. Neurosci. 2, 704–716. 10.1038/35094565 11584308

[B19] Federmeier K. D. (2007). Thinking ahead: the role and roots of prediction in language comprehension. Psychophysiology 44, 491–505. 10.1111/j.1469-8986.2007.00531.x 17521377 PMC2712632

[B20] Federmeier K. D. Kutas M. (1999). A rose by any other name: long-term memory structure and sentence processing. J. Mem. Lang. 41, 469–495. 10.1006/jmla.1999.2660

[B21] Federmeier K. D. Wlotko E. W. De Ochoa-Dewald E. Kutas M. (2007). Multiple effects of sentential constraint on word processing. Brain Res. 1146, 75–84. 10.1016/j.brainres.2006.06.101 16901469 PMC2704150

[B22] Friederici A. D. (2002). Towards a neural basis of auditory sentence processing. Trends Cogn. Sci. 6, 78–84. 10.1016/s1364-6613(00)01839-8 15866191

[B23] Friederici A. D. (2011). The brain basis of language processing: from structure to function. Physiol. Rev. 91, 1357–1392. 10.1152/physrev.00006.2011 22013214

[B24] Friederici A. D. Frisch S. (2000). Verb argument structure processing: the role of verb-specific and argument-specific information. J. Mem. Lang. 43, 476–507. 10.1006/jmla.2000.2709

[B25] Friston K. (2003). Learning and inference in the brain. Neural Netw. 16, 1325–1352. 10.1016/j.neunet.2003.06.005 14622888

[B26] Fruchter J. Linzen T. Westerlund M. Marantz A. (2015). Lexical preactivation in basic linguistic phrases. J. Cogn. Neurosci. 27, 1912–1935. 10.1162/jocn_a_00822 25961637

[B27] Gagnepain P. Henson R. N. Davis M. H. (2012). Temporal predictive codes for spoken words in auditory cortex. Curr. Biol. 22, 615–621. 10.1016/j.cub.2012.02.015 22425155 PMC3405519

[B28] Gramfort A. Luessi M. Larson E. Engemann D. A. Strohmeier D. Brodbeck C.. (2013). MEG and EEG data analysis with MNE-Python. Front. Neurosci. 7:267. 10.3389/fnins.2013.00267 24431986 PMC3872725

[B29] Gramfort A. Luessi M. Larson E. Engemann D. A. Strohmeier D. Brodbeck C.. (2014). MNE software for processing MEG and EEG data. Neuroimage 86, 446–460. 10.1016/j.neuroimage.2013.10.027 24161808 PMC3930851

[B30] Griffiths T. L. Tenenbaum J. B. (2011). Predicting the future as Bayesian inference: people combine prior knowledge with observations when estimating duration and extent. J. Exp. Psychol. Gen. 140, 725–743. 10.1037/a0024899 21875247

[B31] Gunter T. C. Friederici A. D. Schriefers H. (2000). Syntactic gender and semantic expectancy: ERPs reveal early autonomy and late interaction. J. Cogn. Neurosci. 12, 556–568. 10.1162/089892900562336 10936910

[B32] Hald L. A. Steenbeek-Planting E. G. Hagoort P. (2007). The interaction of discourse context and world knowledge in online sentence comprehension. Evidence from the N400. Brain Res. 1146, 210–218. 10.1016/j.brainres.2007.02.054 17433893

[B33] Hale J. (2001). “A probabilistic Earley parser as a psycholinguistic model,” in Proceedings of the Second Meeting of the North American Chapter of the Association for Computational Linguistics on Language Technologies, (Stroudsburg, PA: Association for Computational Linguistics). 1–8

[B34] Halgren E. Dhond R. P. Christensen N. Van Petten C. Marinkovic K. Lewine J. D.. (2002). N400-like magnetoencephalography responses modulated by semantic context, word frequency and lexical class in sentences. Neuroimage 17, 1101–1116. 10.1006/nimg.2002.1268 12414253

[B35] Halgren E. Wang C. Schomer D. L. Knake S. Marinkovic K. Wu J.. (2006). Processing stages underlying word recognition in the anteroventral temporal lobe. Neuroimage 30, 1401–1413. 10.1016/j.neuroimage.2005.10.053 16488158 PMC1513618

[B36] Hämäläinen M. S. Ilmoniemi R. J. (1994). Interpreting magnetic-fields of the brain–minimum norm estimates. Med. Biol. Eng. Comput. 32, 35–42. 10.1007/bf02512476 8182960

[B37] Hickok G. (2012). The cortical organization of speech processing: feedback control and predictive coding the context of a dual-stream model. J. Commun. Disord. 45, 393–402. 10.1016/j.jcomdis.2012.06.004 22766458 PMC3468690

[B38] Huang Y. P. Rao R. P. N. (2011). Predictive coding. Wiley Interdiscip. Rev. Cogn. Sci. 2, 580–593. 10.1002/wcs.142 26302308

[B39] Jakuszeit M. Kotz S. A. Hasting A. S. (2013). Generating predictions: lesion evidence on the role of left inferior frontal cortex in rapid syntactic analysis. Cortex 49, 2861–2874. 10.1016/j.cortex.2013.05.014 23890826

[B40] Kiebel S. J. Daunizeau J. Friston K. J. (2009). Perception and hierarchical dynamics. Front. Neuroinform. 3:20. 10.3389/neuro.11.020.2009 19649171 PMC2718783

[B41] Kutas M. Federmeier K. D. (2000). Electrophysiology reveals semantic memory use in language comprehension. Trends Cogn. Sci. 4, 463–470. 10.1016/s1364-6613(00)01560-6 11115760

[B43] Kutas M. Federmeier K. D. (2011). Thirty years and counting: finding meaning in the N400 component of the event-related brain potential (ERP). Annu. Rev. Psychol. 62, 621–647. 10.1146/annurev.psych.093008.131123 20809790 PMC4052444

[B44] Kutas M. Hillyard S. A. (1980). Reading senseless sentences–brain potentials reflect semantic incongruity. Science 207, 203–205. 10.1126/science.7350657 7350657

[B45] Kutas M. Hillyard S. A. (1984). Brain potentials during reading reflect word expectancy and semantic association. Nature 307, 161–163. 10.1038/307161a0 6690995

[B46] Kutas M. Hillyard S. A. (1989). An electrophysiological probe of incidental semantic association. J. Cogn. Neurosci. 1, 38–49. 10.1162/jocn.1989.1.1.38 23968409

[B47] Landauer T. K. Dumais S. T. (1997). A solution to Plato’s problem: the latent semantic analysis theory of acquisition, induction and representation of knowledge. Psychol. Rev. 104, 211–240. 10.1037/0033-295x.104.2.211

[B48] Lau E. F. Almeida D. Hines P. C. Poeppel D. (2009). A lexical basis for N400 context effects: evidence from MEG. Brain Lang. 111, 161–172. 10.1016/j.bandl.2009.08.007 19815267 PMC2783912

[B49] Lau E. F. Gramfort A. Hämäläinen M. S. Kuperberg G. R. (2013a). Automatic semantic facilitation in anterior temporal cortex revealed through multimodal neuroimaging. J. Neurosci. 33, 17174–17181. 10.1523/JNEUROSCI.1018-13.2013 24155321 PMC3807034

[B50] Lau E. F. Holcomb P. J. Kuperberg G. R. (2013b). Dissociating N400 effects of prediction from association in single-word contexts. J. Cogn. Neurosci. 25, 484–502. 10.1162/jocn_a_00328 23163410 PMC3657387

[B51] Lau E. F. Phillips C. Poeppel D. (2008). A cortical network for semantics: (de)constructing the N400. Nat. Rev. Neurosci. 9, 920–933. 10.1038/nrn2532 19020511

[B52] Lau E. F. Weber K. Gramfort A. Hämäläinen M. S. Kuperberg G. R. (2016). Spatiotemporal signatures of lexical-semantic prediction. Cereb. Cortex 26, 1377–1387. 10.1093/cercor/bhu219 25316341 PMC4785937

[B53] Levy R. (2008). Expectation-based syntactic comprehension. Cognition 106, 1126–1177. 10.1016/j.cognition.2007.05.006 17662975

[B54] Lewis A. G. Bastiaansen M. (2015). A predictive coding framework for rapid neural dynamics during sentence-level language comprehension. Cortex 68, 155–168. 10.1016/j.cortex.2015.02.014 25840879

[B55] Lewis A. G. Schoffelen J. M. Schriefers H. Bastiaansen M. (2016). A predictive coding perspective on beta oscillations during sentence-level language comprehension. Front. Hum. Neurosci. 10:85. 10.3389/fnhum.2016.00085 26973500 PMC4776303

[B56] Li X. Shu H. Liu Y. Li P. (2006). Mental representation of verb meaning: behavioral and electrophysiological evidence. J. Cogn. Neurosci. 18, 1774–1787. 10.1162/jocn.2006.18.10.1774 17014380

[B57] Maess B. Herrmann C. S. Hahne A. Nakamura A. Friederici A. D. (2006). Localizing the distributed language network responsible for the N400 measured by MEG during auditory sentence processing. Brain Res. 1096, 163–172. 10.1016/j.brainres.2006.04.037 16769041

[B58] Marinkovic K. Dhond R. P. Dale A. M. Glessner M. Carr V. Halgren E. (2003). Spatiotemporal dynamics of modality-specific and supramodal word processing. Neuron 38, 487–497. 10.1016/s0896-6273(03)00197-1 12741994 PMC3746792

[B59] Maris E. Oostenveld R. (2007). Nonparametric statistical testing of EEG- and MEG-data. J. Neurosci. Methods 164, 177–190. 10.1016/j.jneumeth.2007.03.024 17517438

[B60] Meyer P. Mecklinger A. Grunwald T. Fell J. Elger C. E. Friederici A. D. (2005). Language processing within the human medial temporal lobe. Hippocampus 15, 451–459. 10.1002/hipo.20070 15714509

[B61] Obleser J. Kotz S. A. (2010). Expectancy constraints in degraded speech modulate the language comprehension network. Cereb. Cortex 20, 633–640. 10.1093/cercor/bhp128 19561061

[B62] Obleser J. Kotz S. A. (2011). Multiple brain signatures of integration in the comprehension of degraded speech. Neuroimage 55, 713–723. 10.1016/j.neuroimage.2010.12.020 21172443

[B63] Oldfield R. C. (1971). The assessment and analysis of handedness: the Edinburgh inventory. Neuropsychologia 9, 97–113. 10.1016/0028-3932(71)90067-4 5146491

[B64] Park H. Ince R. A. Schyns P. G. Thut G. Gross J. (2015). Frontal top-down signals increase coupling of auditory low-frequency oscillations to continuous speech in human listeners. Curr. Biol. 25, 1649–1653. 10.1016/j.cub.2015.04.049 26028433 PMC4503802

[B65] Pascual-Marqui R. D. (2002). Standardized low-resolution brain electromagnetic tomography (sLORETA): technical details. Methods Find. Exp. Clin. Pharmacol. 24, 5–12. 12575463

[B66] Patterson K. Nestor P. J. Rogers T. T. (2007). Where do you know what you know? The representation of semantic knowledge in the human brain. Nat. Rev. Neurosci. 8, 976–987. 10.1038/nrn2277 18026167

[B67] Poeppel D. Idsardi W. J. van Wassenhove V. (2008). Speech perception at the interface of neurobiology and linguistics. Philos. Trans. R. Soc. Lond. B Biol. Sci. 363, 1071–1086. 10.1098/rstb.2007.2160 17890189 PMC2606797

[B68] Price C. J. (2010). The anatomy of language: a review of 100 fMRI studies published in 2009. Ann. N Y Acad. Sci. 1191, 62–88. 10.1111/j.1749-6632.2010.05444.x 20392276

[B69] Pulvermüller F. Shtyrov Y. Ilmoniemi R. (2005). Brain signatures of meaning access in action word recognition. J. Cogn. Neurosci. 17, 884–892. 10.1162/0898929054021111 15969907

[B70] Pylkkänen L. Marantz A. (2003). Tracking the time course of word recognition with MEG. Trends Cogn. Sci. 7, 187–189. 10.1016/s1364-6613(03)00092-5 12757816

[B71] Pylkkänen L. McElree B. (2007). An MEG study of silent meaning. J. Cogn. Neurosci. 19, 1905–1921. 10.1162/jocn.2007.19.11.1905 17958491

[B72] Pylkkänen L. Stringfellow A. Marantz A. (2002). Neuromagnetic evidence for the timing of lexical activation: an MEG component sensitive to phonotactic probability but not to neighborhood density. Brain Lang. 81, 666–678. 10.1006/brln.2001.2555 12081430

[B73] Rao R. P. N. Ballard D. H. (1999). Predictive coding in the visual cortex: a functional interpretation of some extra-classical receptive-field effects. Nat. Neurosci. 2, 79–87. 10.1038/4580 10195184

[B74] Rauss K. Schwartz S. Pourtois G. (2011). Top-down effects on early visual processing in humans: a predictive coding framework. Neurosci. Biobehav. Rev. 35, 1237–1253. 10.1016/j.neubiorev.2010.12.011 21185860

[B75] Salmelin R. (2007). Clinical neurophysiology of language: the MEG approach. Clin. Neurophysiol. 118, 237–254. 10.1016/j.clinph.2006.07.316 17008126

[B76] Schiffer A.-M. Ahlheim C. Wurm M. F. Schubotz R. I. (2012). Surprised at all the entropy: hippocampal, caudate and midbrain contributions to learning from prediction errors. PLoS One 7:e36445. 10.1371/journal.pone.0036445 22570715 PMC3343024

[B77] Shapiro L. P. Gordon B. Hack N. Killackey J. (1993). Verb-argument structure processing in complex sentences in Broca′ s and Wernicke′ s aphasia. Brain Lang. 45, 423–447. 10.1006/brln.1993.1053 8269333

[B78] Shapiro L. P. Zurif E. Grimshaw J. (1987). Sentence processing and the mental representation of verbs. Cognition 27, 219–246. 10.1016/s0010-0277(87)80010-0 3691026

[B79] Smith N. J. Levy R. (2013). The effect of word predictability on reading time is logarithmic. Cognition 128, 302–319. 10.1016/j.cognition.2013.02.013 23747651 PMC3709001

[B80] Sohoglu E. Peelle J. E. Carlyon R. P. Davis M. H. (2012). Predictive top-down integration of prior knowledge during speech perception. J. Neurosci. 32, 8443–8453. 10.1523/JNEUROSCI.5069-11.2012 22723684 PMC6620994

[B81] Strauß A. Kotz S. A. Obleser J. (2013). Narrowed expectancies under degraded speech: revisiting the N400. J. Cogn. Neurosci. 25, 1383–1395. 10.1162/jocn_a_00389 23489145

[B82] Taulu S. Simola J. Kajola M. Helle L. Ahonen A. Sarvas J. (2012). “Suppression of uncorrelated sensor noise and artifacts in multi-channel MEG data,” in 18th International Conference on Biomagnetism, Paris, 285.

[B83] Taulu S. Simola J. Kajola M. (2005). Applications of the signal space separation method. IEEE Trans. Sign. Proc. 53, 3359–3372. 10.1109/tsp.2005.853302

[B84] Taylor W. L. (1953). “Cloze Procedure”: a new tool for measuring readability. Journal. Q. 30, 415–433.

[B85] Thompson C. K. Bonakdarpour B. Fix S. C. Blumenfeld H. K. Parrish T. B. Gitelman D. R.. (2007). Neural correlates of verb argument structure processing. J. Cogn. Neurosci. 19, 1753–1767. 10.1162/jocn.2007.19.11.1753 17958479 PMC2253656

[B86] Thompson-Schill S. L. D’Esposito M. Aguirre G. K. Farah M. J. (1997). Role of left inferior prefrontal cortex in retrieval of semantic knowledge: a reevaluation. Proc. Natl. Acad. Sci. U S A 94, 14792–14797. 10.1073/pnas.94.26.14792 9405692 PMC25116

[B87] Thompson-Schill S. L. D’Esposito M. Kan I. P. (1999). Effects of repetition and competition on activity in left prefrontal cortex during word generation. Neuron 23, 513–522. 10.1016/s0896-6273(00)80804-1 10433263

[B88] Tracy J. I. Boswell S. B. (2008). “Mesial temporal lobe epilepsy: a model for understanding the relationship between language and memory,” in Handbook of the Neuroscience of Language, eds Stemmer B. Whitaker H. A. (Elsevier: UK), 319–328. 10.1016/b978-008045352-1.00031-8

[B90] Van Petten C. Coulson S. Rubin S. Plante E. Parks M. (1999). Time course of word identification and semantic integration in spoken language. J. Exp. Psychol. Learn. Mem. Cogn. 25, 394–417. 10.1037/0278-7393.25.2.394 10093207

[B89] Van Petten C. Luka B. J. (2012). Prediction during language comprehension: benefits, costs and ERP components. Int. J. Psychophysiol. 83, 176–190. 10.1016/j.ijpsycho.2011.09.015 22019481

[B91] Wahl M. Marzinzik F. Friederici A. D. Hahne A. Kupsch A. Schneider G. H.. (2008). The human thalamus processes syntactic and semantic language violations. Neuron 59, 695–707. 10.1016/j.neuron.2008.07.011 18786354

[B92] Wang L. Zhu Z. Bastiaansen M. (2012). Integration or predictability? A further specification of the functional role of gamma oscillations in language comprehension. Front. Psychol. 3:187. 10.3389/fpsyg.2012.00187 22701443 PMC3372880

[B93] Wlotko E. W. Federmeier K. D. (2012). So that’s what you meant! Event-related potentials reveal multiple aspects of context use during construction of message-level meaning. Neuroimage 62, 356–366. 10.1016/j.neuroimage.2012.04.054 22565202 PMC3457057

